# Supplementary material for: Mutations in PCYT2 disrupt etherlipid biosynthesis and cause a complex hereditary spastic paraplegia
Source: Brain. 2019 Oct 22;142(11):3382–97. doi: 10.1093/brain/awz291 (PMC6821184; doi:10.1093/brain/awz291)
Supplement: awz291_Supplementary_Data [file awz291_supplementary_data.zip › awz291-Suppl_data/Supplementary_Data4.zip!/Supplementary information 4 - plasma lipidomics.zip!/Plasma lipidomics/Control vs Patient2/Statistical plots and data/Correlation matrix_AOE.pdf]

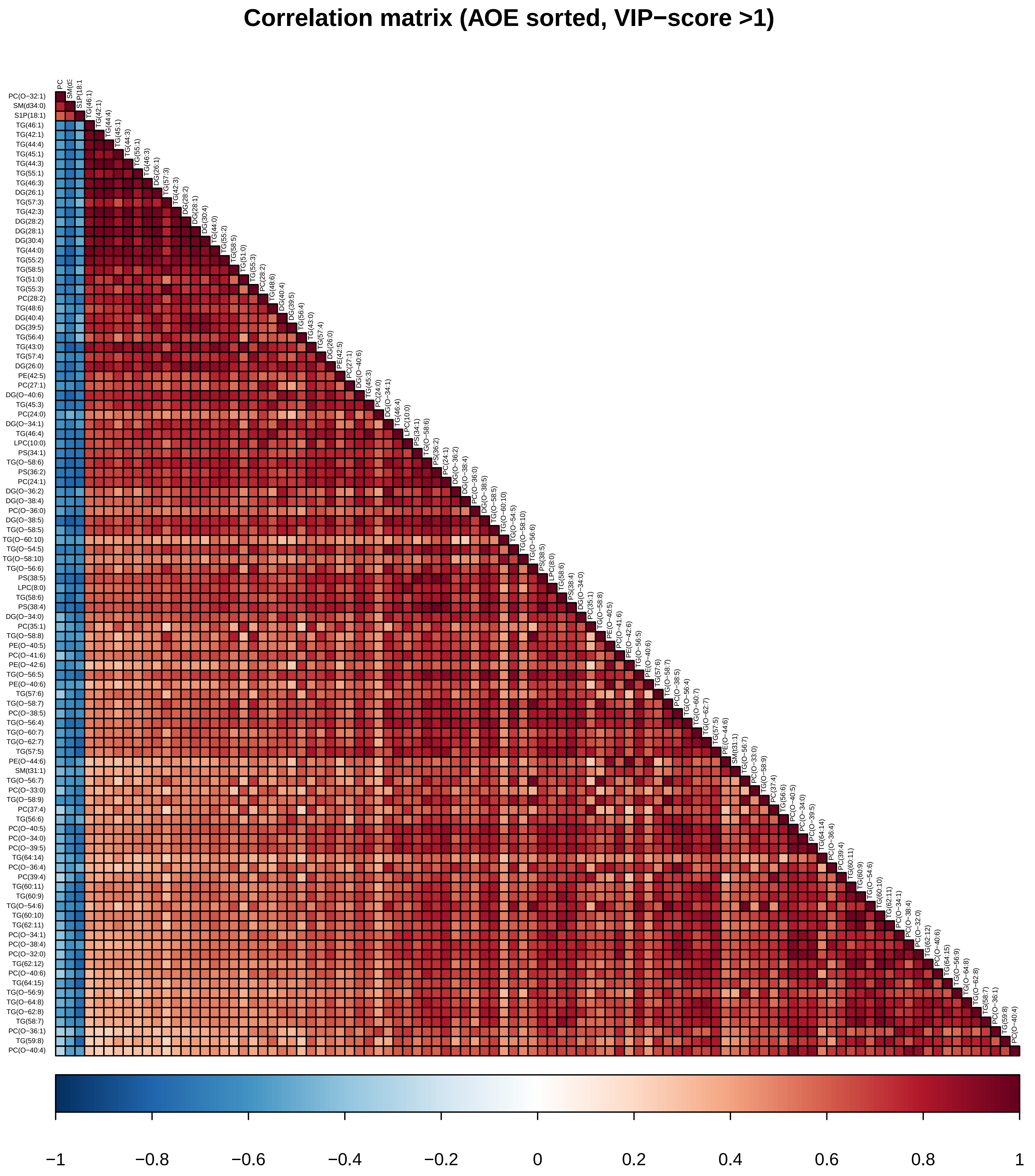
Correlation matrix (AOE sorted, VIP−score >1)
PC(O−32:1)
SM(d34:0)
S1P(18:1)
TG(46:1)
TG(42:1)
TG(44:4)
TG(45:1)
TG(44:3)
TG(55:1)
TG(46:3)
DG(26:1)
TG(57:3)
TG(42:3)
DG(28:2)
DG(28:1)
DG(30:4)
TG(44:0)
TG(55:2)
TG(58:5)
TG(51:0)
TG(55:3)
PC(28:2)
TG(48:6)
DG(40:4)
DG(39:5)
TG(56:4)
TG(43:0)
TG(57:4)
DG(26:0)
PE(42:5)
PC(27:1)
DG(O−40:6)
TG(45:3)
PC(24:0)
DG(O−34:1)
TG(46:4)
LPC(10:0)
PS(34:1)
TG(O−58:6)
PS(36:2)
PC(24:1)
DG(O−36:2)
DG(O−38:4)
PC(O−36:0)
DG(O−38:5)
TG(O−58:5)
TG(O−60:10)
TG(O−54:5)
TG(O−58:10)
TG(O−56:6)
PS(38:5)
LPC(8:0)
TG(58:6)
PS(38:4)
DG(O−34:0)
PC(35:1)
TG(O−58:8)
PE(O−40:5)
PC(O−41:6)
PE(O−42:6)
TG(O−56:5)
PE(O−40:6)
TG(57:6)
TG(O−58:7)
PC(O−38:5)
TG(O−56:4)
TG(O−60:7)
TG(O−62:7)
TG(57:5)
PE(O−44:6)
SM(t31:1)
TG(O−56:7)
PC(O−33:0)
TG(O−58:9)
PC(37:4)
TG(56:6)
PC(O−40:5)
PC(O−34:0)
PC(O−39:5)
TG(64:14)
PC(O−36:4)
PC(39:4)
TG(60:11)
TG(60:9)
TG(O−54:6)
TG(60:10)
TG(62:11)
PC(O−34:1)
PC(O−38:4)
PC(O−32:0)
TG(62:12)
PC(O−40:6)
TG(64:15)
TG(O−56:9)
TG(O−64:8)
TG(O−62:8)
TG(58:7)
PC(O−36:1)
TG(59:8)
PC(O−40:4)
−1
−0.8
−0.6
−0.4
−0.2
0
0.2
0.4
0.6
0.8
1
